# Predictors of Progression in Albuminuria in the General Population: Results from the PREVEND Cohort

**DOI:** 10.1371/journal.pone.0061119

**Published:** 2013-05-27

**Authors:** Lieneke Scheven, Nynke Halbesma, Paul E. de Jong, Dick de Zeeuw, Stephan J. L. Bakker, Ron T. Gansevoort

**Affiliations:** 1 Division of Nephrology, Dept. Internal Medicine, University Medical Center Groningen (UMCG), University of Groningen, Groningen, The Netherlands; 2 Dept of Clinical Pharmacology, University Medical Center Groningen (UMCG), University of Groningen, Groningen, The Netherlands; 3 Dept. of Clinical Epidemiology, Leiden University Medical Center (LUMC), Leiden, The Netherlands; University of Sao Paulo Medical School, Brazil

## Abstract

**Background:**

Urinary albumin excretion is known to be independently associated with progression of renal and cardiovascular disease. The aim of this study was to identify predictors for progression in albuminuria in the general population.

**Methods:**

Data were used of the first 4 screening rounds of a community-based prospective cohort study (PREVEND). Included were 5,825 subjects that at baseline had no known renal disease or macroalbuminuria. Subjects were defined as having progressive albuminuria when they belonged to the quintile of subjects with highest absolute increase in urinary albumin excretion per year and a urinary albumin excretion during the last screening in which they participated of ≥150 mg/24 h. Change in urinary albumin excretion per year was calculated as last available urinary albumin excretion minus baseline UAE divided by follow-up time.

**Results:**

During 9.3 years follow-up 132 subjects had progressive albuminuria. These subjects were significantly older, more often of male gender and had a worse cardiovascular risk profile. In a multivariable model, testing baseline values, significant predictors of progressive albuminuria were male gender (OR 2.23; p<0.001), age (OR 1.03; p<0.001), BMI (OR 1.06; p = 0.02) and baseline albuminuria (OR 5.71; p<0.001). Based on these findings a risk score was made to estimate a subject's risk for progressive albuminuria.

**Conclusion:**

A high baseline albuminuria is by far the most important predictor of progressive albuminuria. Thus, screening for baseline albuminuria will be more important than screening for cardiovascular risk factors in order to identify subjects at risk for progressive albuminuria.

## Introduction

Chronic kidney disease (CKD) is defined by an impaired glomerular filtration rate (GFR) or an increased urinary albumin excretion (UAE).[1] Numerous studies have shown that an impaired GFR is associated with a poor cardiovascular [2], [3], but also with a poor renal outcome [4]. Many studies evaluated which factors are associated with progressive GFR decline. Prediction models have been developed to estimate the risk of an individual to develop end-stage renal disease. Some of these prediction models were developed for high risk populations, such as people with known underlying cardiovascular disease [5], or for specific kidney diseases, such as IgA nephropathy [6] and diabetic nephropathy [7]. We recently published a risk score for future eGFR loss in community dwelling subjects using demographic data, as well as data that can be obtained in screening programs [8].

It has been shown that not only GFR, but also a higher UAE is associated with a worse cardiovascular and renal prognosis [2], [4], [9] and that a rise in UAE is particularly associated with risk of poor cardiovascular or renal outcome [10]–[12]. It is therefore of interest to develop also prediction models to estimate the risk of an individual to develop progressive UAE. As yet such risk models are lacking. Furthermore, information on risk factors for an increase in albuminuria are known in patients with diabetes mellitus. However, such information is not available for the general, predominantly non-diabetic population.

In the present study we therefore investigated which factors are associated with progressive albuminuria. Not only baseline characteristics were taken into account, but also short-term changes in parameters like blood glucose and systolic blood pressure. Using the identified risk factors a model was designed to predict who will develop a progressive increase in albuminuria, in analogy to the model we recently designed to predict for each individual the risk to develop progressive eGFR loss [8].

## Patients and Methods

### Study design and population

This study was conducted using data of subjects participating in the Prevention of REnal and Vascular ENd-stage Disease (PREVEND) study. This prospective, population based cohort study investigates the natural course of UAE and its relation with renal and cardiovascular disease. Details of the study protocol have been published elsewhere [13], [14]. In summary, all inhabitants of the city of Groningen aged 28–75 years were sent a questionnaire and a vial to collect a first-morning-void urine sample. Of these subjects, 40,856 responded (47.8%) and returned this vial to a central laboratory for urinary albumin assessment. From these 40,856 subjects the PREVEND cohort was selected with the aim to create a cohort enriched for the presence of albuminuria. After exclusion of subjects with type 1 diabetes mellitus (defined as subjects requiring the use of insulin) and pregnant females (defined by self report), all subjects with a urinary albumin concentration of >10 mg/L (n = 7,768) were invited for the first screening round, and 6,000 participated. Furthermore, a randomly selected control group with a urinary albumin concentration of <10 mg/L (n = 3,394) was also invited, and 2,592 participated. These 8,592 subjects constitute the actual PREVEND cohort and were asked to collect 2 consecutive 24-hour urine samples (baseline screening). The first screening round was completed in 1997–98 by 8,592 participants. Thereafter, participants were invited to visit the outpatient clinic for a medical examination at approximately 3-year intervals. The PREVEND study was approved by the medical ethics committee of the University Medical Center Groningen and conducted in accordance with the guidelines of the Declaration of Helsinki. All participants gave written informed consent.

For the present study, we excluded subjects with at baseline known renal disease (N = 20) or macroalbuminuria (N = 134), and subjects in whom no data on untreated UAE value was available. At baseline there were: N = 112 (1.9%) participants with CKD stage 1 (eGFR>90 ml/min/1.73 m^2^ and UAE 30-300 mg/24 h); N = 395 (6.8%) participants with CKD stage 2 (eGFR 60-89 ml/min/1.73 m^2^ and UAE 30-300 mg/24 h); N = 228 (3.9%) participants with CKD stage 3 (GFR 30–59 mL/min/1.73 m^2^); N = 26 (0.4%) participants with CKD stage 4 (GFR 15–29 mL/min/1.73 m^2^). There were no subjects with stage CKD stage 5 (GFR <15 mL/min/1.73 m^2^).

### Measurements

During each screening round participants filled out a questionnaire on demographics, cardiovascular and renal disease history, smoking status and the use of oral blood pressure, glucose and lipid lowering drugs. Information on drug use was completed with data from community pharmacies [15] (www.iadb.nl). Anthropometrical measurements were performed, and fasting blood samples were taken. Blood pressure was measured in supine position, every minute, for 10 and 8 min, with an automatic device (Dinamap XL Model 9300; Johnson-Johnson Medical, Tampa, FL). Blood pressure is given as the mean of the last two recordings of both visits. Concentrations of total cholesterol and plasma glucose were measured using standard methods. Serum creatinine was measured by dry chemistry (Eastman Kodak, Rochester, New York, USA), with intra-assay coefficient of variation of 0.9% and interassay coefficient of variation of 2.9%. Participants were instructed not to collect urine in case of infectious diseases and to refrain from intensive physical activity during the collection period. Urinary albumin concentration was measured in these fresh urine samples by nephelometry with a threshold of 2.3 mg/L and intra- and interassay coefficients of variation of 2.2 and 2.6%, respectively (BNII; Dade Behring Diagnostic, Marburg, Germany). UAE is given as the mean of the two 24-h urine collections.

### Definitions

Known kidney disease was defined as present or past kidney disease requiring dialysis. Participants were considered as smoking when they stated to have smoked in the year previous to the screening. A cardiovascular disease history was defined as self reported myocardial infarction, percutaneous transluminal coronary angioplasty, coronary artery bypass graft or cerebrovascular accident. Body mass index was calculated as the ratio between weight and the square of height. Hypertension was defined as systolic blood pressure ≥140 mmHg or diastolic blood pressure ≥90 mmHg or use of blood pressure lowering medication according to self report or pharmacy data (JNC-7 definition). Known hypertension was defined as use of blood pressure lowering medication. Hyperlipidemia was defined as a cholesterol level >5.0 mmol/L when a history of cardiovascular disease was present, a cholesterol level of >6.5 mmol/L when a history of cardiovascular disease was absent, or use of lipid lowering drugs. Known hyperlipidemia was defined as use of lipid lowering medication. Diabetes mellitus was defined as a fasting glucose level of >7.0 mmol/L, a non-fasting glucose level of >11.1 mmol/L or use of glucose lowering medication (ADA definition).[16] Known diabetes was defined as use of glucose lowering medication. eGFR was estimated using the Modification of Diet in Renal Disease (MDRD) study equation, taking into account gender, age, race and serum creatinine. Short-term change in potential risk factors was defined as the difference between values obtained at the second minus the first screening round (median follow-up of 4.2 years).

Subjects were defined as having progressive albuminuria when they belonged to the quintile of subjects with highest *absolute* increase in UAE per year AND a UAE during the last screening in which they participated of ≥150 mg/24 h. Change in UAE per year was calculated as last available UAE minus baseline UAE divided by follow-up time. Of note, in case participants started after the baseline screening medication known to influence the natural course of albuminuria (i.e. blood pressure, glucose or lipid lowering drugs) the last available UAE value before start of such medication was used for analyses. When subjects were already using such medication during the baseline screening, these subjects were eligible.

### Statistical analysis

All calculations were performed with SPSS version 18.0 software. Continuous data are reported as mean ±SD. In case of skewed distribution the median with interquartile range are presented. Differences between the two cohorts for continuous data were tested by Student's t-test or a Mann–Whitney U test in case of skewed distribution. Differences between groups for proportions were tested with a chi-square test.

### Model development

As possible predictors for progressive albuminuria we used baseline parameters, that have been suggested in literature to be renal risk factors: age, gender, cardiovascular disease history, smoking, BMI, systolic blood pressure, use of ACE inhibitor/ Angiotensin Receptor Blocker (ACEi/ARB), known hypertension, total plasma cholesterol, known hyperlipidemia, glucose, known diabetes, eGFR and UAE. First, we analyzed univariable associations between these predictors and progressive albuminuria using logistic regression analysis. Second, a multivariable model was built using backward selection. Only variables having a p-value <0.2 in univariable analysis were included in the multivariable model (model 1). Values of UAE were logarithmically transformed to fulfil the requirement of linearity of the logit. Given the definition of progressive albuminuria it could be expected that baseline albuminuria itself will to be a strong predictor. Therefore also a multivariable model was made excluding baseline albuminuria (model 2). Subsequently, we analysed the association of short-term changes in renal risk factors with progressive albuminuria when added to models 1 and 2 (models 3 and 4, respectively). Model 1 was chosen as final regression model, and tested for possible significant interactions (all possible ones based on variables in model 1) and non-linear associations between continuous predictors and progression in albuminuria by adding quadratic terms. A variable was considered to be statistically significantly associated with progression in albuminuria when p<0.05. Lastly, based on model 1, a score chart for prediction of progressive albuminuria was developed.

### Model validation

The performance of the final model was evaluated by analysis of the area under the Receiver Operating Characteristic (ROC) curve. The internal validity of the prediction model was evaluated by bootstrapping.[17] Two hundred samples of equal size were drawn at random and with replacement from the complete dataset. In these bootstrap samples the coefficients of the final regression model were estimated and tested in the original sample. The slope index (differences between the coefficients in the original sample and bootstrap samples) was used as a shrinkage method by multiplying coefficients with the slope index to correct for ‘optimism’.

### Prediction of an individual subject's risk

A prediction model was developed based on the regression coefficients in the final regression model. With this prediction rule the probability of having progressive albuminuria after a follow-up period of 9.3 years was estimated for each individual. The general equation for estimating the probability (P) of having progressive albuminuria is:
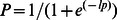



The linear predictor (l p) consists of the regression coefficients estimated in the final model, multiplied by the values of each predictor for each patient. To facilitate calculation of an individual subject's risk in clinical practice analyses were also performed with the predictors of the final prediction model subdivided into clinically meaningful categories. With this model a numerical score chart was derived, by rounding up the estimates of the corresponding regression parameters obtained from the model. The performance of this model was also evaluated by analysis of area under the ROC curve. The diagnostic characteristics of this model in terms of sensitivity, specificity, positive predictive value and negative predictive value were calculated. All analyses were performed with SPSS version 18.0 software, except bootstrapping, which was performed in R version 2.13.0 for Windows.

### Sensitivity analyses

Various sensitivity analyses were performed. First, we used the CKD-EPI equation instead of the MDRD equation to estimate GFR. Second, we used a different definition for progressive albuminuria: an increase in UAE category (normoalbuminuria defined as <30 mg/24 h; microalbuminuria defined as 30-300 mg/24 h and macroalbuminuria defined as >300 mg/24 h) AND doubling of UAE from baseline till last follow-up. Third, we performed a sensitivity analysis with the albumin to creatinine ratio instead of the UAE for defining progression in albuminuria. Finally, the PREVEND cohort was enriched for subjects with higher UAE to acquire sufficient subjects with microalbuminuria. Therefore design-based analyses were performed, which takes into account this study design and allow to draw conclusions that are valid for the general population.

## Results

**Figure 1:**
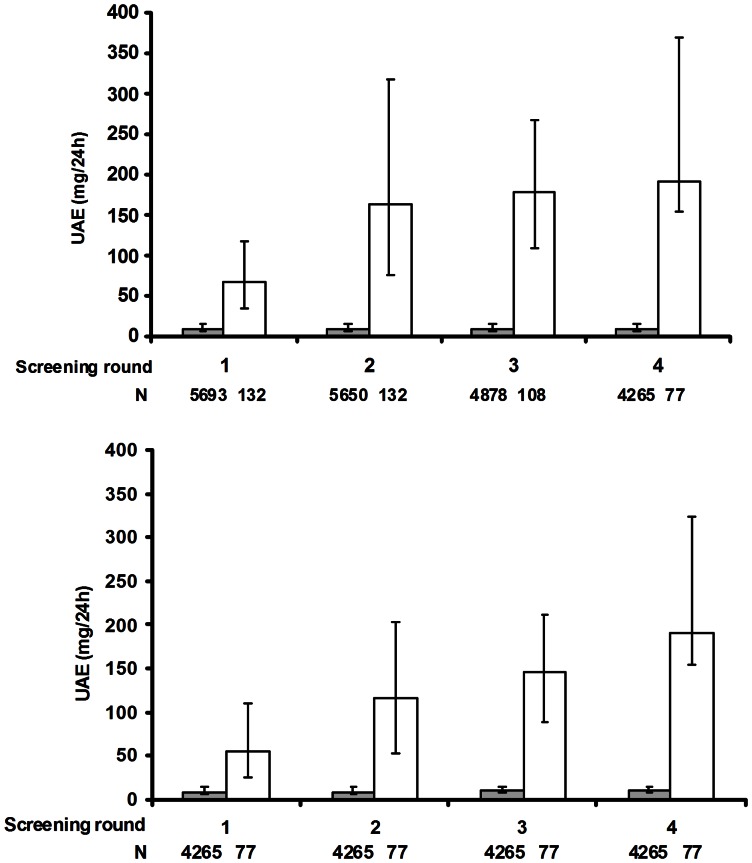
Median (IQR) urinary albumin excretion (UAE) of subjects meeting the definition of progressive albuminuria (open bars) and subjects without progressive albuminuria (dark bars) during the various screening rounds of the PREVEND cohort. The upper panel shows data of all participants, whereas the lower panel shows data of only participants with follow-up until the last screening. Abbreviations: N denotes the number of participants.

From the 8.592 subjects of the PREVEND cohort, we excluded 154 subjects with at baseline known renal disease or macroalbuminuria, and 2613 subjects in whom no follow-up data on untreated UAE was available (1687 subjects with no UAE measurement at follow-up and 926 subjects who started between the baseline and the second screening treatment known to influence the natural course of albuminuria). The present study includes therefore 5,825 subjects. In these subjects median follow-up was 9.3 years (minimum of 3.6 years and maximum of 11.3 years), and 132 subjects met our definition of progressive UAE. Figure 1 shows median UAE values of these subjects during the 4 screening rounds. Seventy seven participants were defined to have progressive UAE based on data obtained at baseline (first) and the last (fourth) screening round. Thirty one participants were defined as such based on data obtained at baseline and the third screening round (no data available at the fourth screening round, or having started blood pressure, glucose or lipid lowering drugs between the third and fourth screening round), and 24 participants based on data obtained at baseline and the second screening round (no data available at the third screening round, or having started blood pressure, glucose or lipid lowering drugs between the second and third screening round). In line with our definition UAE increased gradually during follow-up in these subjects, whereas in other subjects albuminuria remained fairly stable.

Table 1 shows the baseline characteristics of the 5,825 subjects included in this analysis, subdivided according progressive UAE status. The subjects who met the definition of having progressive UAE were at baseline significantly older (57.8 vs. 48.1 years), predominantly male (75.0%) and had a worse cardiovascular risk profile (e.g. higher BMI, systolic blood pressure, diastolic blood pressure and glucose).

**Table 1:** Baseline characteristics.

	Progressive albuminuria	No progressive albuminuria	p-value
Number	132	5693	–
Male (%)	75.0	48.3	<0.001
Age (yrs)	57.8±11.4	48.1±11.9	<0.001
Smoking (%)	40.9	36.1	0.25
History of CVD (%)	12.1	3.7	<0.001
BMI (kg/m^2^)	28.1±4.3	25.6±3.9	<0.001
SBP (mmHg)	138.2±20.7	125.1±17.4	<0.001
DBP (mmHg)	77.8 ±9.4	72.4±8.9	<0.001
Known hypertension (%)	40.0	13.8	<0.001
Use of ACEi or ARB (%)	15.4	4.9	<0.001
Hypertension (%)	61.4	13.8	<0.001
Cholesterol (mmol/L)	5.7±1.1	5.5±1.1	0.09
Known hyperlipidemia (%)	17.4	5.8	<0.001
Hyperlipidemia (%)	43.2	24.1	<0.001
Glucose (mmol/L)	5.1±1.0	4.7±0.8	0.002
Known diabetes (%)	2.3	1.2	0.22
Diabetes (%)	2.3	1.8	0.69
CRP (mg/L)	1.6[0.9–4.0]	1.1[0.5–2.6]	<0.001
Serum creatinine (µmol/L)	94.6±24.6	82.8±13.5	<0.001
eGFR (mL/min/1.73 m^2^)	–	81.4±13.6	<0.001
UAE (mg/24 h)	67.4[35.3-116.4]	8.4[6.1–13.5]	<0.001

Abbreviations: CVD, cardiovascular disease; BMI, body mass index; SBP, systolic blood pressure; DBP, diastolic blood pressure; ACEi, angiotensin converting enzyme inhibitor; ARB, angiotensin receptor blocker; eGFR, estimated glomerular filtration rate; UAE, urinary albumin excretion.

### Model development

In univariable logistic regression analyses age, male gender, cardiovascular disease history, BMI, systolic blood pressure, known hypertension, use of ACEi/ARB, glucose, total cholesterol, known hyperlipidemia, and UAE at baseline were all positively associated with progressive UAE, whereas eGFR showed a negative association with progressive albuminuria. Changes in systolic blood pressure and in glucose of baseline to second screening were also associated with progressive albuminuria (Table 2).

**Table 2:** Results of univariable logistic regression analyses exploring subject characteristics associated with progressive albuminuria.

	OR	95% CI	p-value
Age (yr)	1.07	1.05–1.08	<0.001
Male gender (vs. female)	3.21	2.16–4.77	<0.001
Smoking (y/n)	1.23	0.86–1.74	0.25
Cardiovascular disease history (y/n)	3.55	2.07–6.09	<0.001
BMI (kg/m^2^)	1.12	1.09–1.16	<0.001
SBP (mmHg)	1.03	1.03–1.04	<0.001
Known hypertension (y/n)	3.25	2.18-4.85	<0.001
ACEi/ARB (y/n)	3.51	2.09–5.90	<0.001
Glucose (mmol/L)	1.42	1.24–1.62	<0.001
Known diabetes (y/n)	1.89	0.59–6.08	0.29
Total cholesterol (mmol/L)	1.15	0.98–1.34	0.09
Known hyperlipidemia (y/n)	3.40	2.14–5.41	<0.001
CRP (mg/L)	1.02	0.99–1.05	0.09
Baseline eGFR (mL/min/1.73 m^2^)	0.97	0.95–0.98	<0.001
UAE (mg/24 h), ln-transformed	6.34	5.22–7.71	<0.001
Change in SBP (mmHg)	1.01	0.99–1.03	0.07
Change in cholesterol (mmol/L)	0.94	0.75–1.18	0.57
Change in BMI (kg/m^2^)	0.99	0.88–1.10	0.79
Change in glucose (mmol/L)	1.23	1.07–1.40	0.003

Abbreviations: BMI, body mass index; SBP, systolic blood pressure; ACEi, angiotensin converting enzyme inhibitor; ARB, angiotensin receptor blocker; eGFR, estimated glomerular filtration rate; UAE, urinary albumin excretion; OR, odds ratio; CI, confidence interval.

Table 3 presents the various multivariable models. Odds ratios, p-values and Wald-statistics are only shown for variables that, after backward selection, contributed to the model with a p<0.05. In model 1, only including baseline covariates, male gender, age, BMI and baseline UAE were associated with progressive albuminuria. In model 2, the model with similar baseline covariates but baseline UAE deleted from the model, the same covariates as in model 1 were associated with progressive albuminuria, and in addition it was found that systolic blood pressure, eGFR and known hyperlipidemia were significantly associated with progressive albuminuria. Models 3 and 4 are similar to models 1 and 2, respectively, but include in addition changes in covariates from baseline to second screening. Model 3 showed similar results as model 1, but now also an increase in systolic blood pressure was associated with progressive albuminuria. When excluding UAE, model 4 shows results essentially similar to model 2, whereas in addition an increase in glucose and an increase in systolic blood pressure were associated with progressive albuminuria. Of note, there were no interaction terms between variables that remained in model 1, which showed a significant association with progressive albuminuria.

**Table 3:** Results of the multivariable logistic regression analyses exploring subject's characteristics associated with progressive albuminuria.

Model 1				Model 2			Model 3			Model 4		
R^2^ 0.41				R2 0.14			R2 0.42			R2 0.15		
OR (95% CI)		p-value	Wald	OR (95% CI)	p-value	Wald	OR (95% CI)	p-value	Wald	OR (95% CI)	p-value	Wald
Male (vs. female)	2.23 (1.02–1.05)	0.001	11.9	3.43 (2.24–5.25)	<0.001	32.3	2.26 (1.43–3.58)	<0.001	12.2	3.45 (2.23–5.33)	<0.001	31.2
Age (yrs)	1.03 (1.02–1.05)	<0.001	13.7	1.04 (1.02–1.06)	<0.001	16.4	1.03 (1.02–1.05)	<0.001	13.1	1.03 (1.01–1.05)	0.004	8.2
Smoking (y/n)												
History of CVD												
Body Mass Index (kg/m^2^)	1.06 (1.01–1.11)	0.02	5.6	1.11 (1.06-1.16)	<0.001	22.5	1.06 (1.01–1.12)	0.01	6.0	1.10 (1.05–1.15)	<0.001	18.1
SBP (mmHg)				1.01 (1.00–1.02)	0.04	4.1				1.01 (1.00–1.03)	0.02	5.7
Known hypertension (y/n)										1.69 (1.10–2.59)	0.02	5.8
Use of ACEi or ARB (y/n)												
Cholesterol (mmol/L)												
Known hyperlipidemia (y/n)				1.80 (1.11–2.91)	0.02	5.7						
Glucose (mmol/L)												
Known diabetes (y/n)												
CRP (mg/L)												
eGFR (mL/min/1.73m^2^)				0.98 (0.97–0.99)	0.01	6.7				0.98 (0.96–-0.99)	0.005	8.1
UAE, ln-transformed	5.71 (4.64–7.02)	<0.001	273.3	NA	NA	NA	5.78 (4.70–7.11)	<0.001	275.3	NA	NA	NA
Change in BMI (kg/m^2^)	NA	NA	NA	NA	NA	NA						
Change in glucose (mmol/L)	NA	NA	NA	NA	NA	NA				1.17 (1.03–1.34)	0.02	5.8
Change in SBP (mmHg)	NA	NA	NA	NA	NA	NA	1.02 (1.01–1.03)	0.006	7.4	1.02 (1.00–1.03)	0.01	6.5
Change in cholesterol (mmol/L)	NA	NA	NA	NA	NA	NA						

Abbreviations: CVD, cardiovascular disease; SBP, systolic blood pressure; ACEi, angiotensin converting enzyme inhibitor; ARB, angiotensin receptor blocker; eGFR, estimated glomerular filtration rate; UAE, urinary albumin excretion; BMI, body mass index; OR, odds ratio; NA, not applicable.

### Model validation

The multivariable regression coefficients of our final model (Table 3, model 1) were additionally multiplied with the shrinkage factor (0.99) that was obtained after bootstrapping, to correct for ‘optimism’. Based on these optimism corrected coefficients of the multivariable logistic regression model presented in Table 3, the prediction rule as given in equation 1 was constructed. When making a ROC curve of model 1, our final model, the area under the ROC curve was 0.94 [95%CI 0.92 to 0.96], indicating that the discrimination of the model is high.
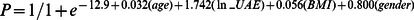



Equation 1 is the Prediction rule based on the optimism corrected coefficients of the multivariable logistic regression model. In Equation 1 age is entered in years, gender as being male (1) or female (0); body mass index as kg/m^2^ and albuminuria as mg/24 h.

### Prediction of an individual subject's risk

Subsequently, for clinical use a model was built with the variables that significantly contributed to the final model (model 1, Table 3) subdivided into clinically meaningful categories. Results are given in Table 4, which shows the numbers of subjects per covariate, subdivided into meaningful categories and the odds ratio's with the lowest category as reference. A higher baseline UAE was most markedly associated with a higher risk to develop progressive albuminuria. Having an UAE of 15–30 mg/24 h compared to <15 mg/24 h had a greater odds ratio than being >70 years of age, male, or being obese. The importance of baseline UAE to predict progressive albuminuria is also reflected by the R-square of the full model, which differ between 0.41 when baseline UAE was included and 0.14 when baseline UAE was excluded.

**Table 4:** Results of the univariable and multivariable logistic regression analyses for progressive albuminuria.

Variable	N (total)	N (progressors)	OR (univariable) (95% CI)	OR (multivariable) (95% CI)
**Age (yrs)**				
<50 (ref)	3448	42 (1.2%)	1.00	1.00
50–70	2089	71 (3.4%)	2.85 (1.94–4.20)	1.20 (0.77–1.88)
>70	288	19 (6.6%)	5.73 (3.29–9.99)	1.74 (0.91–3.30)
Gender				
Male	2851	99 (3.5%)	3.21 (2.16–4.77)	2.23 (1.43-3.47)
**BMI (kg/m^2^)**				
<18.5	40	0 (0%)	0.00 (−)	0.00 (−)
18.5–24 (ref)	2678	29 (1.1%)	1.00	1.00
25–29	2300	67 (2.9%)	2.74 (1.77–4.25)	1.64 (1.01-2.68)
>30	744	36 (4.8%)	4.65 (2.83–7.63)	2.02 (1.16-3.51)
**UAE (mg/**24 h**)**				
<15 (ref)	4489	15 (0.3%)	1.00	1.00
15–29	769	13 (1.7%)	5.13 (2.43–10.8)	4.29 (2.02–9.11)
30–149	520	81 (15.6%)	55.0 (31.4–96.3)	41.7 (23.4–74.3)
150–300	47	23 (48.9%)	285.8 (133.1–613.8)	233.0 (105.9–512.4)

Abbreviations: BMI, body mass index; UAE, urinary albumin excretion; SBP, systolic blood pressure; N, number; OR, odds ratio.

The regression coefficients of this model were also corrected for ‘optimism’ by multiplying the coefficients of the original model by the shrinkage factor (shrinkage = 0.98). Based on the coefficients of the model in Table 4 a score chart was derived that is presented in Table 5. It shows that the predominant covariate to predict progressive albuminuria is baseline albuminuria. For instance, a woman of <50 years with a BMI <25 kg/m^2^, but with a baseline UAE of 30–150 mg/24 h scores 17 points, which is more than the 10 points that are scored by an obese man of >70 years, but with a baseline UAE<15 mg/24 h. The area under the ROC curve was 0.92 [95%CI 0.89 to 0.94], indicating that the discrimination of the model is high. The diagnostic characteristics of the model are given in Table 6.

**Table 5:** Score chart.

Score chart	
Characteristic	Points
**Age (yrs)**	
<50 (reference)	0
50–70	1
>70	3
**Gender**	
Male	4
**Body mass index (kg/m^2^)**	
<18.5	0
18.5-25 (reference)	0
25-30	2
>30	3
**Albuminuria (mg/**24 h**)**	
<15 (reference)	0
15-30	6
30-150	17
150-300	24
**Total score**	**Risk (%)**
<16	0-5%
17-20	5–10%
21-23	10–20%
24-25	20–30%
26-27	30–40%
28-29	40–50%
30-31	–
32-33	60-70%
34	>70

**Table 6:** Diagnostic characteristics of the prediction model.

Total score	Population (%)	Identified events (%)	Sensitivity (%)	Specificity (%)	PPV	NPV
≥30	0.3 (17)	9.8 (13)	9.8	99.9	76.5	98.0
≥29	0.4 (23)	12.1 (16)	12.1	99.9	69.6	98.0
≥28	0.5 (29)	12.1 (16)	12.1	99.9	55.2	98.0
≥27	0.9 (52)	15.9 (21)	15.9	99.5	40.4	98.1
≥26	1.4 (81)	22.0 (29)	22.0	99.1	35.8	98.2
≥25	2.1 (121)	32.6 (43)	32.6	98.6	35.5	98.4
≥24	4.4 (254)	50.0 (66)	50.0	96.7	26.0	98.8
≥23	5.2 (300)	54.5 (72)	54.5	96.0	24.0	98.9
≥22	5.9 (340)	59.8 (79)	59.8	95.4	23.2	99.1
≥21	7.2 (415)	69.7 (92)	69.7	94.3	22.2	99.3
≥20	8.0 (461)	73.5 (97)	73.5	93.6	21.0	99.3
≥15	10.3 (593)	79.5 (105)	79.5	91.4	17.7	99.5
≥10	18.0 (1037)	87.9 (116)	87.9	83.7	11.2	99.9
≥5	48.1 (2772)	97.0 (128)	97.0	53.1	4.6	99.9
≥0	100.0 (5762)	100.0 (132)	100.0	0.0	2.3	100.0

Abbreviations. PVV, positive predicting value; NPV, negative predicting value.

### Sensitivity analyses

Various sensitivity analyses were performed. First, we used the CKD-EPI equation [18] instead of the MDRD equation to estimate GFR. It showed that model 1, which we adopted to use as our final model, presents results similar to our primary analyses ( Table S1). Second, we used a different definition for progressive albuminuria: an increase in UAE category and doubling of UAE from baseline till last follow-up. By using this definition 362 subjects were defined as having progressive albuminuria. Their baseline characteristics are shown in Table S2. Baseline median UAE level and albuminuria during follow-up (16.3 [10.7–23.0] mg/24 h) and 56.4 (40.8-106.5), respectively) were less compared to the value obtained in the 132 progressors that met the definition of progressive albuminuria adopted for the primary analyses. Like the final model of the primary analyses, the final model of this sensitivity analysis showed that baseline UAE, age, male gender, BMI and known hyperlipidemia were significantly associated with progressive albuminuria (Table S3). Third, when performing a sensitivity analysis defining progressive albuminuria using albumin to creatinine ratio instead of urinary albumin excretion 104 participants were defined as having progressive UAE. Only minor differences were observed in the multivariable regression models when compared to the primary analyses (eGFR instead of age included in the final model, Table S4. Finally, because of the enrichment of the PREVEND study for higher levels of albuminuria we performed weighted analyses. Again similar results were obtained as in the primary analyses, the only difference being that now also known hypertension and use of ACE inhibitors/ angiotensin receptor blockers were significant covariates.

## Discussion

In the present study we show that in a community-based cohort a higher baseline UAE is the most important variable that predicts whether an individual will develop progressive albuminuria during follow-up. Of all other variables taken into account only higher age, male gender and higher BMI were predictors for the risk to develop progressive albuminuria, but not higher systolic blood pressure and higher plasma glucose. Systolic blood pressure only became a significant predictor when we eliminated baseline UAE from the multivariable model. When we, in addition, took into account also changes in baseline covariates, it appeared that also changes in systolic blood pressure and plasma glucose were predictors for progressive albuminuria. Finally, we found that when building a score chart to predict risk for progressive albuminuria, the risk associated with a baseline UAE in the range of microalbuminuria by far outweighed the risk associated with male gender, a higher baseline age or BMI.

Thus far, most studies investigating factors that are associated with progression of UAE have been performed in subjects with type 1 or type 2 diabetes mellitus [19]–[22] Baseline UAE, male gender, blood pressure and HbA1c concentration were independently associated with progression of albuminuria in a cohort of Caucasian subjects with type 1 diabetes [21]. Similarly, in a Japanese cohort with subjects with type 2 diabetes under tight glycemic and blood pressure control, baseline UAE and systolic blood pressure were positively correlated with progression of UAE [22]. Only few studies investigated predictors of progressive albuminuria in non-diabetic populations. A recent analysis of the ONTARGET study that was performed in subjects at high risk for vascular disease (of which about two third was non-diabetic) showed with multivariable regression analysis, that an increase in systolic blood pressure and in heart rate and a fall in serum creatinine were associated with changes in UAE [11]. Another study performed in the Framingham Offspring Cohort baseline UAE, age, male gender, diabetes mellitus, smoking, and low LDL cholesterol were associated with incident microalbuminuria [23]. In general, these data are in line with our present findings obtained in a cohort of community dwelling subjects with only few diabetic patients included. Our data show that progressive albuminuria is also observed in non-diabetic subjects, and that the factors associated with progressive albuminuria in non-diabetics show great overlap with those in diabetics. Interestingly, the impact of having progressive albuminuria may also be comparable in non-diabetic and diabetic subjects. In non-diabetic subjects progressive albuminuria has also been shown to be associated with an increased risk for cardiovascular morbidity and mortality [11], [12], [24], and for the need for dialysis [11].

How could this score chart be implemented in clinical practice? This chart suggests that if someone has a modestly elevated UAE, even in absence of other renal risk factors, follow up of UAE may be indicated. The impact of baseline albuminuria is very strong when compared to the impact of the other predictors. The score chart shows the relative impact of the various factors. The scoring system will be of limited help in clinical assessment.

It is surprising that the predictive value of baseline UAE is that important that blood pressure, plasma glucose and changes in these covariates became only significant predictors of progressive albuminuria after omitting baseline UAE from the multivariable models. This finding can be of help for our understanding of the interaction between blood pressure, glucose and UAE. Traditionally, it was argued that elevated glucose and blood pressure are the driving forces for a rise in UAE. This concept was however challenged when it was shown in a population survey that the prevalence of microalbuminuria in subjects not known to have diabetes or hypertension is relatively high, being 6.6% [25]. In comparison, microalbuminuria was found in 11.5% of those known with hypertension, and in 16.4% of those known with diabetes [25]. Since in the general population there are far more subjects without diabetes mellitus or hypertension, than there are subjects with diabetes mellitus or hypertension, this implied that in the majority of microalbuminuric subjects microalbuminuria was not due to these risk factors [25]. In line with this observation, it was later shown that microalbuminuria is frequent not the result of diabetes mellitus or hypertension, but that microalbuminuria may also precede the onset of these risk factors [26]–[29]. Interestingly, it has recently been hypothesized that at birth one is endowed with a level of UAE, representing a vascular state that is determined genetically or due to in-utero environmental factors, and that this level of UAE may be associated with susceptibility to organ damage [30], [31]. This would explain why albuminuria is an independent predictor of cardiovascular and renal outcome, and new-onset hypertension and diabetes. Our present findings of a predominant role of baseline UAE to predict progressive albuminuria could be interpreted to support this hypothesis.

Our findings on risk prediction for progressive albuminuria are in line with previous findings from the same cohort on risk prediction for progressive renal function loss [8]. In both studies we used a similar approach to define cases. In our study of predictors of progressive renal function loss baseline eGFR was also the most important predictor, in analogy of baseline albuminuria predicting progressive albuminuria. In the prediction model for eGFR loss, however, other factors, such as baseline systolic blood pressure, UAE and C-reactive protein were also significantly and strongly associated with risk, whereas in the present prediction model for progressive albuminuria the relative contribution of these risk factors is limited. Insights in the factors that predict progression of albuminuria is the more relevant as a rise in UAE has been shown to often precede a fall in eGFR, especially in type 1 [32] and type 2 [33] diabetes mellitus. As moreover, changes in eGFR in the range from normal to 60 ml/min/1.73 m^2^ are relative difficult to detect, in these early cases follow up of changes in UAE may be warranted to check for progression in chronic kidney disease.

Our study has some limitations that need to be mentioned. First, the data are limited to a Caucasian population, and thus the results cannot directly be extrapolated to other ethnic groups. Second, some variables under investigation were based on self-report, which may have led to misclassification. In the present study, however, questionnaire data were combined with objective data, retrieved from pharmacy databases (medication use) or quantitatively measured (glucose and blood pressure). Third, as the intra-individual level of UAE is subject to variation, e.g. due to physical activity and inflammatory diseases, misclassification may have been occurred. If this would be relevant, it will however only strengthen our main conclusion since this is expected to lead to an under- rather than overestimation of the significance of baseline UAE as predictor of progressive albuminuria. Moreover, special care was taken to assess UAE as precise as possible, for participants were advised not to collect urine in case of infectious diseases and to refrain from intensive physical activity during the urine collection period. Fourth, one should consider whether our definition of progressive albuminuria is appropriate. We choose a definition that combined a relative (belonging to the quintile of subjects with highest percentage increase in UAE per year from baseline to end of follow-up) AND an absolute criterion (UAE at end of follow-up >150 mg/24 h) in analogy to the definition we used to study a risk prediction model for progressive eGFR loss [8] and in analogy to literature [4]. Importantly, our sensitivity analyses using other criteria to define progressive albuminuria showed essentially similar results. Fifth, the PREVEND cohort is enriched for subjects with higher levels of UAE. We therefore performed weighted analyses to adjust for this oversampling, which rendered essentially similar results. Furthermore, because of exclusion of participants without data on albuminuria at the second screening round (and no data during follow-up), with subsequently no possible calculation of progression in albuminuria, any selection bias could have been possible. Furthermore, since this study used internal validation, other studies are needed to test external validity. Furthermore, ACEi/ARB use at baseline was included as potential indicator of risk for progression of albuminuria. We concluded that indeed the use of this medication was associated with higher risk. This probably reflects bias by indication, i.e. that subjects with a high chance for progressive albuminuria were at baseline more likely to use this medication that is known from literature to have a special favorable effect on albuminuria. Finally, participants were censored in case they started blood pressure, glucose and/or lipid lowering medication. This was done because we wanted to study the natural course of albuminuria and this medication is known to influence albuminuria (i.e. causing a decrease). Censoring these participants after the second screening will not form a problem for our analyses, because in these subjects follow-up albuminuria is available before start of this medication. These subjects are therefore eligible to be defined as having “progressive albuminuria”. Only in subjects that started such medication between the baseline and second screening this medication there is no follow-up information on albuminuria available. This indeed might be a source of bias.

What is the result when patients with CKD were excluded? In our analyses we did not exclude subjects with a baseline UAE of 30–300 mg/24 h (N = 566), as it has been shown that the cut-off for microalbuminuria, that is >30 mg/24 h, is highly arbitrary. The risk associated with albuminuria is continuous, with subjects having UAE values as low as 10 mg/24 h already having a higher risk than those with <10 mg/24 h. When we exclude participants with baseline CKD (defined as baseline eGFR <60 mL/min/1.73 m^2^ and/or baseline albuminuria> 30 mg/24 h) there are only 16 subjects left that meet our definition of progression in albuminuria for further analyses. This is not surprising as we defined our endpoint not only as change in UAE, but also as that final UAE should be at least 150 mg/24 h. This definition ensures that the change in UAE is also clinically relevant. When limiting the population to subjects with a baseline UAE value <30 mg/24 h, it will take them longer to reach the value >150 mg/24 h. Consequently only a low number of subjects will have reached this endpoint during the follow-up in our study. With a number of 16 “cases” multivariable regression analyses are not possible.

Strengths of our study is that our data were obtained in a relatively large scale epidemiological study in community dwelling individuals with serial follow-up, that was specifically designed to study the natural course of albuminuria. As such our data are not post-hoc findings, but hypothesis driven. Data of four subsequent screening rounds are available, with detailed objective information on many covariates, including medication use. Furthermore, albuminuria was assessed at each screening in two 24 h urine samples, whereas in most epidemiological studies albuminuria is assessed in one random spot sample, which is known to be subject to more variability [34]. This makes the PREVEND cohort uniquely suited to study the natural course of UAE and to investigate risk factors for an increase in UAE in the general population.

In conclusion, baseline albuminuria is by far the most important variable that predicts risk of progressive albuminuria. It outweighs other factors, such as age, male gender, body mass index, blood pressure and glucose. Thus, in case screening programs are to be designed to identify subjects at risk for progressive albuminuria and associated morbidity and mortality, screening for albuminuria is of more importance than screening for other cardiovascular risk factors. Moreover, our data suggest that if someone has a modestly elevated UAE, even in absence of other cardiovascular risk factors, follow-up of albuminuria may be indicated.

## Supporting Information

Table S1 **Results of the multivariable logistic regression analyses for progressive albuminuria.** GFR estimated with the CKD-EPI equation instead of the MDRD equation.Abbreviations: CVD, cardiovascular disease; BMI, body mass index; SBP, systolic blood pressure; DBP, diastolic blood pressure; ACEi, angiotensin converting enzyme inhibitor; ARB, angiotensin receptor blocker; eGFR (CKD-EPI), estimated glomerular filtration rate; UAE, urinary albumin excretion; OR, odds ratio; NA, not applicable.
(DOC)

Table S2 **Baseline characteristics for subjects with and without progressive albuminuria.** Progressive UAE defined as an increase in UAE category and doubling of UAE from baseline until last follow-up.Abbreviations: CVD, cardiovascular disease; BMI, body mass index; SBP, systolic blood pressure; DBP, diastolic blood pressure; ACEi, angiotensin converting enzyme inhibitor; ARB, angiotensin receptor blocker; eGFR, estimated glomerular filtration rate; UAE, urinary albumin excretion.
(DOC)

Table S3 **Results of the multivariable logistic regression analyses exploring subject characteristics associated with progressive albuminuria.** Progressive albuminuria defined as an increase in albuminuria category and doubling of albuminuria from baseline until last follow-up.Abbreviations: CVD, cardiovascular disease;; SBP, systolic blood pressure; ACEi, angiotensin converting enzyme inhibitor; ARB, angiotensin receptor blocker; eGFR, estimated glomerular filtration rate; UAE, urinary albumin excretion; BMI, body mass index; OR, odds ratio; NA, not applicable.
(DOC)

Table S4 **Results of the multivariable logistic regression analyses exploring subject’s characteristics associated with progressive albuminuria, based on the albumin to creatinine ratio.** Abbreviations: CVD, cardiovascular disease; SBP, systolic blood pressure; ACEi, angiotensin converting enzyme inhibitor; ARB, angiotensin receptor blocker; eGFR, estimated glomerular filtration rate; UAE, urinary albumin excretion; BMI, body mass index; OR, odds ratio; NA, not applicable.
(DOC)
