# Supplementary material for: Predictors of Progression in Albuminuria in the General Population: Results from the PREVEND Cohort
Source: PLoS One. 2013 May 27;8(5):e61119. doi: 10.1371/journal.pone.0061119 (PMC3664562; doi:10.1371/journal.pone.0061119)
Supplement: Table S1 — Results of the multivariable logistic regression analyses for progressive albuminuria. GFR estimated with the CKD-EPI equation instead of the MDRD equation.Abbreviations: CVD, cardiovascular disease; BMI, body mass index; SBP, systolic blood pressure; DBP, diastolic blood pressure; ACEi, angiotensin converting enzyme inhibitor; ARB, angiotensin receptor blocker; eGFR (CKD-EPI), estimated glomerular filtration rate; UAE, urinary albumin excretion; OR, odds ratio; NA, not applicable. (DOC) [file pone.0061119.s001.doc]

**Table S1.** Results of the multivariable logistic regression analyses for progressive albuminuria. GFR estimated with the CKD-EPI equation instead of the MDRD equation.

|  | **Model 1** |  |  | **Model 2** |  |  | **Model 3** |  |  | **Model 4** |  |  |
| --- | --- | --- | --- | --- | --- | --- | --- | --- | --- | --- | --- | --- |
|  | **R^2^ 0.41** |  |  | **R^2^ 0.14** |  |  | **R^2^ 0.42** |  |  | **R^2^ 0.15** |  |  |
|  | **OR (95% CI)** | **p-value** | **Wald** | **OR (95% CI)** | **p-value** | **Wald** | **OR (95% CI)** | **p-value** | **Wald** | **OR (95% CI)** | **p-value** | **Wald** |
| Male (vs. female) | 2.23 (1.41-3.51) | 0.001 | 11.9 | 3.30 (2.17-5.03) | <0.001 | 31.0 | 2.26 (1.43-3.58) | <0.001 | 12.2 | 3.43 (2.23-5.28) | <0.001 | 31.3 |
| Age (yrs) | 1.03 (1.02-1.05) | <0.001 | 13.7 | 1.03 (1.01-1.05) | 0.001 | 10.2 | 1.03 (1.02-1.05) | <0.001 | 13.1 | 1.03 (1.01-1.05) | 0.001 | 11.8 |
| Smoking (y/n) |  |  |  |  |  |  |  |  |  |  |  |  |
| History of CVD |  |  |  |  |  |  |  |  |  |  |  |  |
| Body Mass Index (kg/m^2^) | 1.06 (1.01-1.11) | 0.02 | 5.61 | 1.11 (1.07-1.16) | <0.001 | 22.7 | 1.06 (1.01-1.12) | 0.01 | 5.98 | 1.11 (1.06-1.16) | <0.001 | 19.7 |
| SBP (mmHg) |  |  |  | 1.01 (1.00-1.02) | 0.05 | 3.94 |  |  |  | 1.02 (1.00-1.03) | 0.006 | 7.55 |
| Known hypertension (y/n) |  |  |  |  |  |  |  |  |  |  |  |  |
| Use of ACEi or ARB (y/n) |  |  |  |  |  |  |  |  |  |  |  |  |
| Cholesterol (mmol/L) |  |  |  |  |  |  |  |  |  |  |  |  |
| Known hyperlipidemia (y/n) |  |  |  | 1.80 (1.11-2.92) | 0.02 | 5.72 |  |  |  | 1.80 (1.10-2.95) | 0.02 | 5.48 |
| Glucose (mmol/L) |  |  |  |  |  |  |  |  |  |  |  |  |
| Known diabetes (y/n) |  |  |  |  |  |  |  |  |  |  |  |  |
| CRP (mg/L) |  |  |  |  |  |  |  |  |  |  |  |  |
| eGFR (mL/min/1.73m^2^) |  |  |  | 0.98 (0.97-0.99) | 0.009 | 6.92 |  |  |  | 0.98 (0.96-0.99) | 0.005 | 7.83 |
| UAE (mg/24h), ln-transformed | 5.71 (4.64-7.02) | <0.001 | 273.3 | NA | NA | NA | 5.78 (4.70-7.11) | <0.001 | 275.3 | NA | NA | NA |
| Change in BMI (kg/m^2^) | NA | NA | NA | NA | NA | NA |  |  |  |  |  |  |
| Change in glucose (mmol/L) | NA | NA | NA | NA | NA | NA |  |  |  | 1.16 (1.02-1.32) | 0.03 | 4.93 |
| Change in SBP (mmHg) | NA | NA | NA | NA | NA | NA | 1.02 (1.01-1.03) | 0.006 | 7.44 | 1.02 (1.00-1.03) | 0.01 | 6.19 |
| Change in cholesterol (mmol/L) | NA | NA | NA | NA | NA | NA |  |  |  |  |  |  |
