# Supplementary material for: Predictors of Progression in Albuminuria in the General Population: Results from the PREVEND Cohort
Source: PLoS One. 2013 May 27;8(5):e61119. doi: 10.1371/journal.pone.0061119 (PMC3664562; doi:10.1371/journal.pone.0061119)
Supplement: Table S2 — Baseline characteristics for subjects with and without progressive albuminuria. Progressive UAE defined as an increase in UAE category and doubling of UAE from baseline until last follow-up.Abbreviations: CVD, cardiovascular disease; BMI, body mass index; SBP, systolic blood pressure; DBP, diastolic blood pressure; ACEi, angiotensin converting enzyme inhibitor; ARB, angiotensin receptor blocker; eGFR, estimated glomerular filtration rate; UAE, urinary albumin excretion. (DOC) [file pone.0061119.s002.doc]

**Table S2**. Baseline characteristics for subjects with and without progressive albuminuria. Progressive UAE defined as an increase in UAE category and doubling of UAE from baseline until last follow-up.

| **Variables** | **Progressive albuminuria** | **No progressive albuminuria** | **p-value** |
| --- | --- | --- | --- |
| Number | 362 | 5463 | - |
| Male (%) | 65.2 | 47.9 | <0.001 |
| Age (yrs) | 55.4 ±12.0 | 47.9 ±11.8 | <0.001 |
| Smoking (%) | 38.1 | 35.9 | 0.42 |
| History of CVD (%) | 10.5 | 3.5 | <0.001 |
| Body Mass Index (kg/m^2^) | 27.0 ±3.9 | 25.6 ±4.0 | <0.001 |
| SBP (mmHg) | 132.7 ±18.2 | 124.9 ±17.5 | <0.001 |
| DBP (mmHg) | 76.2 ±8.9 | 72.3 ±8.9 | <0.001 |
| Known hypertension (%) | 30.6 | 13.3 | <0.001 |
| Use of ACEi or ARB (%) | 9.9 | 3.8 | <0.001 |
| Hypertension (%) | 48.3 | 24.8 | <0.001 |
| Cholesterol (mmol/L) | 5.6 ±1.0 | 5.5 ±1.1 | 0.05 |
| Known hyperlipidemia (%) | 14.4 | 5.5 | <0.001 |
| Hypercholesterolemia (%) | 44.5 | 35.4 | <0.001 |
| Glucose (mmol/L) | 5.1 ±1.0 | 4.7 ±0.7 | <0.001 |
| Known diabetes (%) | 2.8 | 1.1 | 0.003 |
| Diabetes (%) | 4.1 | 1.6 | 0.001 |
| Serum creatinine (µmol/L) | 87.8 ±20.0 | 82.8 ±13.4 | <0.001 |
| CRP (mg/L) | 1.5 [0.8-3.2] | 1.1 [0.5-2.6] | 0.12 |
| eGFR (mL/min/1.73m^2^) | 78.9 ±15.0 | 81.4 ±13.6 | 0.002 |
| UAE (mg/24h) | 16.3 [10.7-23.0] | 8.3 [6.0-13.2] | <0.001 |
