# Supplementary material for: Predictors of Progression in Albuminuria in the General Population: Results from the PREVEND Cohort
Source: PLoS One. 2013 May 27;8(5):e61119. doi: 10.1371/journal.pone.0061119 (PMC3664562; doi:10.1371/journal.pone.0061119)
Supplement: Table S3 — Results of the multivariable logistic regression analyses exploring subject characteristics associated with progressive albuminuria. Progressive albuminuria defined as an increase in albuminuria category and doubling of albuminuria from baseline until last follow-up.Abbreviations: CVD, cardiovascular disease;; SBP, systolic blood pressure; ACEi, angiotensin converting enzyme inhibitor; ARB, angiotensin receptor blocker; eGFR, estimated glomerular filtration rate; UAE, urinary albumin excretion; BMI, body mass index; OR, odds ratio; NA, not applicable. (DOC) [file pone.0061119.s003.doc]

**Table S3.** Results of the multivariable logistic regression analyses exploring subject characteristics associated with progressive albuminuria. Progressive albuminuria defined as an increase in albuminuria category and doubling of albuminuria from baseline until last follow-up.

|  | **Model 1** |  |  | **Model 2** |  |  | **Model 3** |  |  | **Model 4** |  |  |
| --- | --- | --- | --- | --- | --- | --- | --- | --- | --- | --- | --- | --- |
|  | **R^2^ 0.12** |  |  | **R^2^ 0.09** |  |  | **R^2^ 0.14** |  |  | **R^2^ 0.11** |  |  |
|  | **OR (95% CI)** | **p-value** | **Wald** | **OR (95% CI)** | **p-value** | **Wald** | **OR (95% CI)** | **p-value** | **Wald** | **OR (95% CI)** | **p-value** | **Wald** |
| Male (vs. female) | 1.65 (1.31-2.08) | <0.001 | 18.0 | 1.90 (1.51-2.39) | <0.001 | 30.6 | 1.72 (1.36-2.17) | <0.001 | 20.5 | 1.82 (1.44-2.30) | <0.001 | 25.0 |
| Age (yrs) | 1.04 (1.03-1.05) | <0.001 | 55.3 | 1.04 (1.03-1.05) | <0.001 | 80.7 | 1.03 (1.02-1.04) | <0.001 | 46.6 | 1.04 (1.03- 1.05) | <0.001 | 42.3 |
| Smoking (y/n) |  |  |  |  |  |  |  |  |  |  |  |  |
| History of CVD (y/n) |  |  |  |  |  |  |  |  |  |  |  |  |
| Body Mass Index (kg/m^2^) | 1.04 (1.01-1.06) | 0.02 | 5.9 | 1.05 (1.03-1.05) | <0.001 | 14.5 | 1.04 (1.01-1.07) | 0.01 | 6.7 | 1.04 (1.01-1.07) | 0.008 | 7.1 |
| SBP (mmHg) |  |  |  |  |  |  |  |  |  | 1.01 (1.00-1.02) | 0.023 | 5.2 |
| Known hypertension (y/n) |  |  |  |  |  |  |  |  |  |  |  |  |
| Use of ACEi or ARB (y/n) |  |  |  |  |  |  |  |  |  |  |  |  |
| Cholesterol (mmol/L) |  |  |  |  |  |  |  |  |  |  |  |  |
| Known hyperlipidemia (y/n) | 1.64 (1.17-2.29) | 0.004 | 8.2 | 1.77 (1.27-2.46) | 0.001 | 11.6 | 1.63 (1.16-2.29) | 0.005 | 8.0 | 1.76 (1.26-2.46) | 0.001 | 11.0 |
| Glucose (mmol/L) |  |  |  |  |  |  |  |  |  | 1.13 (1.00-1.27) | 0.05 | 4.0 |
| Known diabetes (y/n) |  |  |  |  |  |  |  |  |  |  |  |  |
| CRP (mg/L) |  |  |  |  |  |  |  |  |  |  |  |  |
| eGFR (mL/min/1.73m^2^) |  |  |  |  |  |  |  |  |  |  |  |  |
| UAE (mg/24h), ln-transformed | 1.71 (1.52-1.93) | <0.001 | 79.4 | NA | NA | NA | 1.77 (1.57-1.99) | <0.001 | 87.1 | NA | NA | NA |
| Change in BMI (kg/m^2^) | NA | NA | NA | NA | NA | NA |  |  |  |  |  |  |
| Change in glucose (mmol/L) | NA | NA | NA | NA | NA | NA |  |  |  |  |  |  |
| Change in SBP (mmHg) | NA | NA | NA | NA | NA | NA | 1.03 (1.02-1.04) | <0.001 | 40.4 | 1.03 (1.02-1.04) | <0.001 | 39.7 |
| Change in cholesterol (mmol/L) | NA | NA | NA | NA | NA | NA |  |  |  |  |  |  |
