# Supplementary material for: Predictors of Progression in Albuminuria in the General Population: Results from the PREVEND Cohort
Source: PLoS One. 2013 May 27;8(5):e61119. doi: 10.1371/journal.pone.0061119 (PMC3664562; doi:10.1371/journal.pone.0061119)
Supplement: Table S4 — Results of the multivariable logistic regression analyses exploring subject’s characteristics associated with progressive albuminuria, based on the albumin to creatinine ratio. Abbreviations: CVD, cardiovascular disease; SBP, systolic blood pressure; ACEi, angiotensin converting enzyme inhibitor; ARB, angiotensin receptor blocker; eGFR, estimated glomerular filtration rate; UAE, urinary albumin excretion; BMI, body mass index; OR, odds ratio; NA, not applicable. (DOC) [file pone.0061119.s004.doc]

**Table S4.** Results of the multivariable logistic regression analyses exploring subject’s characteristics associated with progressive albuminuria, based on the albumin to creatinine ratio.

|  | **Model 1** |  |  | **Model 2** |  |  | **Model 3** |  |  | **Model 4** |  |  |
| --- | --- | --- | --- | --- | --- | --- | --- | --- | --- | --- | --- | --- |
|  | **R^2^ 0.42** |  |  | **R^2^ 0.14** |  |  | **R^2^ 0.43** |  |  | **R^2^ 0.15** |  |  |
|  | **OR (95% CI)** | **p-value** | **Wald** | **OR (95% CI)** | **p-value** | **Wald** | **OR (95% CI)** | **p-value** | **Wald** | **OR (95% CI)** | **p-value** | **Wald** |
| Male (vs. female) | 2.54 (1.56-4.14) | <0.001 | 14.0 | 2.05 (1.33-3.16) | 0.001 | 10.6 | 3.02 (1.87-4.86) | <0.001 | 20.5 | 2.07 (1.34-3.21) | 0.001 | 10.7 |
| Age (yrs) | 1.02 (1.00-1.05) | 0.05 | 3.84 | 1.04 (1.02-1.06) | <0.001 | 14.5 |  |  |  | 1.04 (1.02-1.06) | 0.001 | 11.5 |
| Smoking (y/n) |  |  |  |  |  |  |  |  |  |  |  |  |
| History of CVD (y/n) |  |  |  |  |  |  |  |  |  |  |  |  |
| Body Mass Index (kg/m^2^) | 1.08 (1.03-1.14) | 0.002 | 9.34 | 1.09 (1.04-1.14) | 0.001 | 11.9 | 1.09 (1.04-1.15) | 0.001 | 11.5 | 1.08 (1.03-1.14) | 0.002 | 9.3 |
| SBP (mmHg) |  |  |  | 1.01 (1.00-1.03) | 0.008 | 7.12 |  |  |  | 1.02 (1.01-1.03) | 0.001 | 11.9 |
| Known hypertension (y/n) |  |  |  |  |  |  |  |  |  |  |  |  |
| Use of ACEi or ARB (y/n) |  |  |  |  |  |  |  |  |  |  |  |  |
| Cholesterol (mmol/L) |  |  |  |  |  |  |  |  |  |  |  |  |
| Known hyperlipidemia y/n) |  |  |  | 1.74 (1.02-2.95) | 0.04 |  |  |  |  | 1.82 (1.07-3.10) | 0.03 | 4.91 |
| Glucose (mmol/L) |  |  |  |  |  |  |  |  |  |  |  |  |
| Known diabetes (y/n) |  |  |  |  |  |  |  |  |  |  |  |  |
| CRP (mg/L) |  |  |  |  |  |  |  |  |  |  |  |  |
| eGFR (mL/min/1.73m^2^) | 0.98 (0.96-0.99) | 0.005 | 7.80 | 0.97 (0.96-0.99) | 0.001 | 11.7 | 0.97 (0.95-0.98) | <0.001 | 17.1 | 0.97 (0.96-0.99) | 0.001 | 10.7 |
| ACR (mg/mmol), ln-transformed | 5.73 (4.58-7.18) | <0.001 | 231.7 | NA | NA | NA | 6.09 (4.86-7.64) | <0.001 | 245.1 | NA | NA | NA |
| Change in BMI (kg/m^2^) | NA | NA | NA | NA | NA | NA |  |  |  |  |  |  |
| Change in glucose (mmol/L) | NA | NA | NA | NA | NA | NA |  |  |  | 1.16 (1.00-1.33) | 0.05 | 4.0 |
| Change in SBP (mmHg) | NA | NA | NA | NA | NA | NA | 1.03 (1.01-1.05) | <0.001 | 13.3 | 1.02 (1.01-1.04) | 0.001 | 10.8 |
| Change in cholesterol (mmol/L) | NA | NA | NA | NA | NA | NA |  |  |  |  |  |  |
